# Novel Point Mutations in Frataxin Gene in Iranian Patients with Friedreich’s Ataxia

**Published:** 2014

**Authors:** Mohammad Mehdi HEIDARI, Mehri KHATAMI, Jafar POURAKRAMI

**Affiliations:** 1 Department of Biology, Faculty of Sciences, Yazd University, Yazd, Iran.; 2 Department of Biology, Faculty of Sciences, Science and Research Branch of the Islamic Azad University, Tehran, Iran.

**Keywords:** Friedreich’s ataxia, FXN gene, Mutation, PCR-SSCP

## Abstract

**Objective:**

Friedreich’s ataxia is the most common form of hereditary ataxia with autosomal recessive pattern. More than 96% of patients are homozygous for GAA repeat extension on both alleles in the first intron of FXN gene and the remaining patients have been shown to be heterozygous for a GAA extension in one allele and point mutation in other allele.

**Materials & Methods:**

In this study, exons of 1, 2, 3, and 5 of frataxin gene were searched by single strand conformation polymorphism polymerase chain reaction (PCR-SSCP) in 5 patients with GAA extension in one allele. For detection of exact mutation, samples with band shifts were sent for DNA sequencing.

**Results:**

Three novel point mutations were found in patients heterozygous for the GAA repeat expansion, p.S81A, p.Y123D, and p.S192C.

**Conclusion:**

Our results showed that these point mutations in one allele with GAA extension in another allele are associated with FRDA signs. Thus, these results emphasize the importance of performing molecular genetic analysis for point mutations in FRDA patients.

## Introduction

Friedreich’s ataxia (FRDA) is the most prevalent autosomal recessive hereditary ataxia affecting approximately 1 in 50,000 individuals (1). The essential diagnostic criteria, as defined by Harding (2), comprised of onset before 25 years of age and within 5 years of onset, there is progressive ataxia of limbs and gait, absence of knee and ankle jerks and extensor plantar responses, and dysarthria after 5 years of symptom onset (3).

The majority of FRDA patients is homozygous for GAA repeat expansions within the first intron of the FXN gene (4). In normal alleles, the repeat has variation in size between 6 and 36 GAA, whereas in mutated alleles, the repeat length varies from 120 to 1700 (4-9). In patients who are heterozygous for the expanded allele (2-4%), nucleotide substitutions, small deletions, and insertions are found (10,11). 

We investigated five ataxia patients with heterozygosity for point mutations in FXN gene via PCR-SSCP analyses. In this study, we report three novel point mutations, which have been identified in our patients, one at codon 81 in exon 2 (S81A), the second at codon 123 in exon 3 (Y123D), and the third at codon 192 in exon 5a (S192C).

## Materials & Methods

**Patients**

We investigated 5 patients (3 females and 2 males) heterozygous for the GAA trinucleotide repeat genotype in the FRDA gene with a slowly progressive gait ataxia compatible with autosomal recessive inheritance. Twenty-five healthy controls (15 females and 10 males) matched for age, sex, and ethnicity, were selected. All of the patients and control group were informed on the aims of the study and gave their informed consents for the genetic analysis. Patients were diagnosed and referred for assessment by consultant neurologists.

**Mutation analysis**

Genomic DNA was isolated from whole blood using standard procedures. Repeat expansions were detected by PCR amplification of 250 ng DNA using primers 5200 Eco and 5200 Not, together with the Expand Long Template PCR System (Roche, Mannheim, Germany), which was previously described (12-14). 

The GAA repeat length was calculated based on the size of the PCR product (457+3n bp, n= number of GAA triplets) (15).

**Fig. 1:**
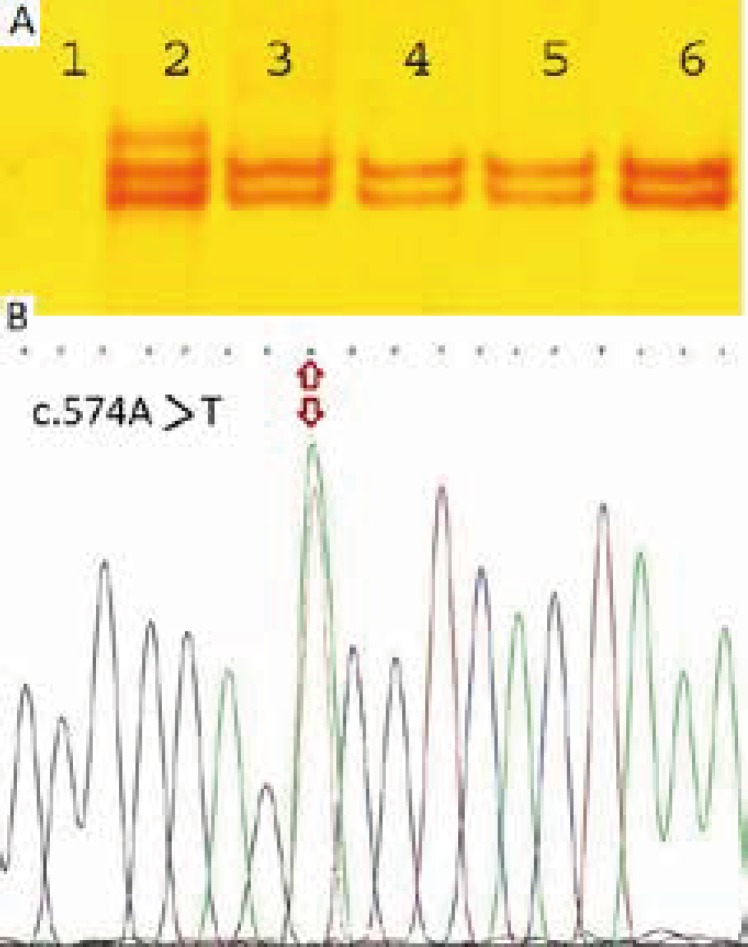
**A) **Single-strand conformation polymorphism (SSCP) analysis of exon 5 amplified products: Lane 1 represents non-denatured DNA; Lane 2 represents the patient’s DNA, showing an abnormal band; lanes 3, 4, 5, and 6 represent normal control DNAs. B) Sequencing result

Single-strand conformational polymorphism (SSCP) analysis was performed on the four main frataxin exons (exons of 1, 2, 3, and 5), including exon–intron boundaries (Table 1). Electrophoresis was done in different conditions of temperature and voltage for each PCR reaction. After electrophoresis, gel was stained with silver staining method (16). The SSCP variant bands were excised from the gel and directly sequenced by automated sequencing 3700 ABI machine (Macrogene Seoul, South Korea) (Fig. 1).

**Table 1:** Primer Sequences and Length Fragments

**Exons**	**Primer Sequences**	**Size (bp)**
1	FRE1F: 5'-AGTCTCCCTTGGGTCAGGGGTCCTGG-3' FRE1R: 5'-CCGCGGCTGTTCCCGG-3'	413
2	FRE2F: 5'-GGCACTCGAATGTAGAAGTAGC-3' FRE2R: 5'-AGAGGAAGATACCTATGACGTG-3'	234
3	FRE3F: 5'-AAAATGGAAGCATTTGGTAATCA-3' FRE3R: 5'-AGTGAACTAAAATTCTTAGAGGG-3'	228
5	FRE5F: 5'-CTGAAGGGCTGTGCTGTGGA-3' FRE5R: 5'-TGTCCTTAAAACGGGGCT-3'	222

**Pathogenicity analysis**

The secondary structural characteristics and physicochemical properties (hydropathy index, flexibility index, and antigenic index) were determined by Protean (protein structure prediction and annotation) and the conservation of the amino acid changes was assayed using MEGALIGN program, which is part of the Lasergene V.6 software (DNASTAR, Inc. Madison, WI). The hedrophathy index was measured by Protean based on the Kyte-Doolitle method (17), which predicts the regional hydropathy of proteins by their amino acid sequence. Hydropathy values were assigned for all amino acids and then were averaged over a window size equal to 9. Results less than 0 are hydrophobic and more than 0 are hydrophilic.

## Results

Molecular analysis was performed for five patients with heterozygosity for the GAA repeat expansion and 25 healthy controls. The size of GAA expansion observed in our compound heterozygous FRDA patients, ranged from 490 to 873.

Three novel point mutations was found (Table 2) in three patients by PCR-SSCP, one at position c.241T>G resulting in the amino-acid exchange p.Ser81Ala, the second at position c.367T>G resulting in p.Tyr186Asp, and the third at position c.574A>T resulting in the aminoacid exchange p. Ser192Cys within frataxin. These sequence alterations were not found in the controls.

**Table 2:** Summary of Mutations Identified In The Study Subjects

Patient	Exon	Mutation	Effect
1	2	c.241T>G	p.S81A
3	5	c.574A>T	p.S192C
4	3	c.367T>G	p.Y123D

## Discussion

FRDA is well defined by clinical criteria, including are flexia and onset before age 25. An inverse correlation was observed by Campuzano et al. between the amount of frataxin protein and the size of the GAA repeat on the smaller allele (15). Previous observations have shown a correlation between the length of the smaller allele and phenotypic severity (6,18). Therefore, the milder clinical presentation in subjects with smaller GAA repeat sizes is probably to be because of higher levels of frataxin present in such patients. Since the GAA expansion changes protein levels rather than function, examination of the few patients having point mutations in their FRDA gene will help to clarify the key functional domains of frataxin. It is supposed that the expanded allele can contribute to the phenotype in patients who are compound heterozygous for an expansion on one allele and a point mutation on the other, but we cannot quantify this contribution. So, although very rare, locus genetic heterogeneity exists in Friedreich’s ataxia. In addition, ataxia with vitamin E deficiency caused by mutations in the a-TTP gene is expressed as a Friedreich-like phenotype (19).

Various point mutations have been associated with milder and atypical phenotypes in heterozygous patients (20-22).

Patient 1 is a male who presented with an onset of symptoms at 9 years of age with asymmetric ataxia. Recent examination showed spinocerebellar ataxia, pes cavus, and reduced vibration sense. He was found to be heterozygous for an expanded FRDA allele of approximately 870 GAA repeats. Using SSCP analysis and sequencing, a point mutation of a T to a G at codon 81 was detected. c.241T>G sequence change is located at codon 81 and alters a moderately conserved serine, a polar amino acid, to alanine, a nonpolar amino acid, altering the hydropathy index from -0.733 to -0.444. 

Patient 3 is also a 22-year-old female. Onset of symptoms was at 14 years with gait disturbance. She had one allele in the expanded range of 620 repeats. SSCP analysis and sequencing revealed a single base substitution of a A to a T altering codon 192. c.574A>T sequence change is located at amino acid position 192. It changes a moderately conserved serine (a hydrophilic amino acid) to cysteine (a hydrophilic amino acid), which changes the hydropathy index from 0.756 to 0.389.

Patient 4 is a Caucasian female, who is currently 24 years old, with an onset of symptoms at 15 years of age with frequent tripping. She has progressively worsening lower limb ataxia and currently uses a walking stick. She was also heterozygous for the expansion in FRDA with an expanded allele of 490 repeats. Sequencing the genomic DNA of this patient revealed a single base substitution of a T to a G at amino acid position 186. c.367T>G sequence variant is located at amino acid position 186 in frataxin gene. It changes a highly conserved thyrosine (a hydrophobic amino acid) to aspartic acid (a hydrophilic amino acid), which changes the hydropathy index from -0.389 to -0.633 (Fig. 1). 

In conclusion, point mutations in FRDA that causes an absence of functional frataxin, are associated with a severe phenotype. Our results showed that these point mutations in one allele and GAA extension in another allele are associated with FRDA signs. Thus, these results emphasize the importance of performing molecular genetic analysis for point mutations in FRDA patients.

**Fig 2:**
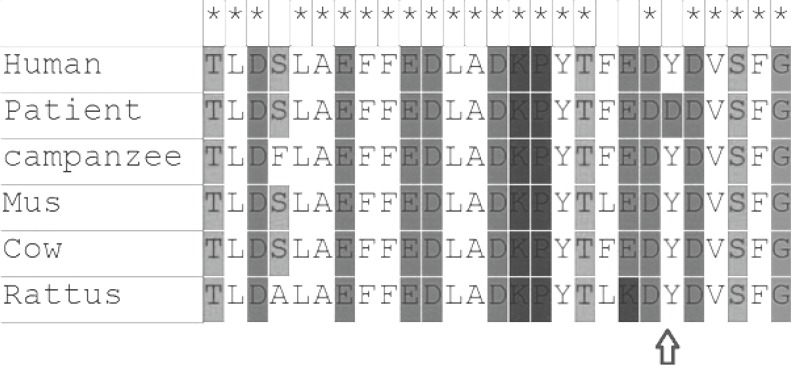
Sequence alignment of frataxin sequences from the human, chimpanzee, mouse, cow, and rattus for p.Y123D mutation in exon 3
